# Draft Genome Sequence of Highly Virulent Race 4/Biovar 3 of *Ralstonia solanacearum* CaRs_Mep Causing Bacterial Wilt in Zingiberaceae Plants in India

**DOI:** 10.1128/genomeA.01420-16

**Published:** 2017-01-05

**Authors:** Aundy Kumar, Vibhuti Munjal, Neelam Sheoran, Thekkan Puthiyaveedu Prameela, Rajamma Suseelabhai, Rashmi Aggarwal, Rakesh Kumar Jain, Santhosh J. Eapen

**Affiliations:** a Division of Plant Pathology, ICAR-Indian Agricultural Research Institute, New Delhi, India; b Division of Crop Protection, ICAR-Indian Institute of Spices Research, Kozhikode, Kerala, India

## Abstract

The genome of *Ralstonia solanacearum* CaRs_Mep, a race 4/biovar 3/phylotype I bacterium causing wilt in small cardamom and other Zingiberaceae plants, was sequenced. Analysis of the 5.7-Mb genome sequence will aid in better understanding of the genetic determinants of host range, host jump, survival, pathogenicity, and virulence of race 4 of *R. solanacearum*.

## GENOME ANNOUNCEMENT

*Ralstonia solanacearum*, causing bacterial wilt, is one of the important plant pathogens, owing to its virulence, aggressiveness, and long survival in environment (1). Five races of *R. solanacearum* have been recorded to infect over 450 plant species, including important vegetables and spices (2). Zingiberaceae plants are affected by race 4/biovar 3 or race 4/biovar 4, belonging to phylotype I clade (2–4). In India, race 4/biovar 3 is primarily responsible for wilt in plants belonging to the Zingiberaceae family (3–6). *Ralstonia solanacearum* CaRs_Mep, a race 4/biovar 3 pathogen, was isolated from bacterial wilt affecting small cardamom plants originating from Meppadi (11.5550°N, 76.1349°E), Wyanad district of the southern Indian state of Kerala (7). Although the genome sequence of race 4/biovar 4 is already published (8), the genome sequence of race 4/biovar 3, the highly virulent lineage of race 4, is not reported yet.

Genomic DNA was extracted from 36-h-old CaRs_Mep isolate using the NucleoSpin tissue DNA isolation kit and quantified using a Qubit fluorometer. The paired-end sequencing library was prepared using the NEBNext Ultra DNA library prep kit for Illumina (Illumina, USA). The quantity and quality of the library were assessed on TapeStation 4200 (Agilent Technologies, USA) using a high-sensitivity D5000 ScreenTape assay kit, as per the manufacturer’s instructions. Whole-genome sequencing data of 2 × 150-bp chemistry were generated on a NextSeq 500. The raw reads generated were filtered using Trimmomatic (version 0.35), with a quality value (QV) >30, and adapters were trimmed. The filtered high-quality reads were assembled into contigs using Velvet (version 1.2.10) on optimized k-mer of 127 (9). The high-quality data obtained were 1.76 Gb with 59,41,389 reads. The obtained primary assembly was further optimized by scaffolding tool SSPACE Basic (version 2.0), where primary assembly (Velvet-produced contigs) and paired-end reads were used (10). High-quality reads were assembled initially using Velvet; as a result, 531 contigs were generated, with an *N*_50_ of 43,135 bp. Initial assembly was further optimized by using SSPACE Basic, which resulted in 253 scaffolds with an *N*_50_ value of 57,727 bp, producing a total assembly length of 5.7 Mb.

Genes were predicted from the assembled scaffolds using Prodigal, with default parameters (11). Functional annotation of the genes was performed using BLASTx program. Gene Ontology (GO) annotations of the genes were determined by the Blast2GO program (12). A total of 5,127 genes were predicted, with sizes ranging from 60 bp to 14,142 bp. The genes were categorized into three main categories, including biological process (2,985 genes), molecular functions (2,813 genes), and cellular components (1,811 genes). A perusal of the records on genome sequences of *R. solanacearum* reveals that this is the first report of a draft genome sequence of race 4/biovar 3. The sequence information presented herein will enable genetic and functional analyses of Zingiberaceae infecting race 4 *R. solanacearum*.

### Accession number(s).

The genome sequence of *R. solanacearum* strain CaRs_Mep has been deposited in the GenBank database under the accession number MCBM00000000. The version described in this paper is MCBM01000000.

## References

[1] Sakthivel K, Kumar A, Devendrakumar C, Vibhuti M, Sheoran N, Gautam RK, Kumar K, Roy SD, Vinatzer BA 2015 Diversity of *Ralstonia solanacearum* strains on the Andaman Islands in India. Plant Dis 100:732–738. doi:10.1094/PDIS-03-15-0258-RE. 30688609

[2] Kumar A, Hayward AC 2005 Bacterial diseases of ginger and their control, p 341–366. *In* Ravindran PN, Babu KN (ed), Monograph on ginger. CRC Press, Boca Raton, FL eBook ISBN: 978-1-4200-2336-7.

[3] Kumar A, Sarma YR 2004 Characterization of *Ralstonia solanacearum* causing bacterial wilt of ginger in India. Indian Phytopathol 57:12–17.

[4] Kumar A, Sarma YR, Anandaraj M 2004 Evaluation genetic diversity of *Ralstonia solanacearum* causing bacterial wilt of ginger using rep-PCR and RFLP-PCR. Curr Sci 87:1555–1561. http://tejas.serc.iisc.ernet.in/currsci/dec102004/1555.pdf.

[5] Kumar A, Prameela TP, Suseelabhai R 2013 A unique DNA repair and recombination gene (*recN*) sequence for identification and intraspecific molecular typing of bacterial wilt pathogen *Ralstonia solanacearum* and its comparative analysis with ribosomal DNA sequences. J Biosci 38:267–278. doi:10.1007/s12038-013-9312-0. 23660661

[6] Kumar A, Prameela TP, Suseelabhai R, Siljo A, Anandaraj M, Vinatzer BA 2014 Host specificity and genetic diversity of race 4 strains of *Ralstonia solanacearum*. Plant Pathol (BSPP) 63:1138–1148. doi:10.1111/ppa.12189.

[7] Kumar A, Prameela TP, Suseelabhai R, Siljo A, Biju CN, Anandaraj M, Vinatzer BA 2011 Small cardamom (*Elettaria cardamomum* Maton) and ginger (*Zingiber officinale* Roxb.) bacterial wilt is caused by same strain of *Ralstonia solanacearum*: a revelation by multilocus sequence typing (MLST). Eur J Plant Pathol 132:477–482. doi:10.1007/s10658-011-9903-2.

[8] Shan W, Yang X, Ma W, Yang Y, Guo X, Guo J, Zheng H, Li G, Xie B 2013 Draft Genome sequence of *Ralstonia solanacearum* race 4 biovar 4 strain SD54. Genome Announc 1(6):e00890-13. doi:10.1128/genomeA.00890-13. 24356823 PMC3868847

[9] Zerbino DR, Birney E 2008 Velvet: algorithms for *de novo* short read assembly using de Bruijn graphs. Genome Res 18:821–829. doi:10.1101/gr.074492.107. 18349386 PMC2336801

[10] Boetzer M, Henkel CV, Jansen HJ, Butler D, Pirovano W 2011 Scaffolding pre-assembled contigs using SSPACE. Bioinformatics 27:578–579. doi:10.1093/bioinformatics/btq683. 21149342

[11] Hyatt D, Chen GL, Locascio PF, Land ML, Larimer FW, Hauser LJ 2010 Prodigal: prokaryotic gene recognition and translation initiation site identification. BMC Bioinformatics 11:119. doi:10.1186/1471-2105-11-119. 20211023 PMC2848648

[12] Götz S, García-Gómez JM, Terol J, Williams TD, Nagaraj SH, Nueda MJ, Robles M, Talón M, Dopazo J, Conesa A 2008 High-throughput functional annotation and data mining with the Blast2GO suite. Nucleic Acids Res 36:3420–3435. doi:10.1093/nar/gkn176. 18445632 PMC2425479

